# Diagnostic accuracy of a commercial AI digital stethoscope for diagnosis of TB

**DOI:** 10.5588/ijtldopen.25.0360

**Published:** 2025-10-10

**Authors:** H. Cox, Y. Rani, L. Nakiyingi, K.A. Francia, Y. Xie, C. Hoang, N. Hapeela, G.P. Romero, E. Nasinghe, N. Van Hung, S. Kim, A. Penn-Nicholson, S.E. Dorman, for the FEND-TB Consortium

**Affiliations:** ^1^ Division of Medical Microbiology and Institute of Infectious Disease and Molecular Medicine, University of Cape Town, Cape Town, South Africa;; ^2^ Burnet Institute, Melbourne, VIC, Australia;; ^3^ Frontier Science Foundation, Brookline, MA, USA;; ^4^ Makerere University, Kampala, Uganda;; ^5^ Universidad Peruana Cayetano Heredia, Lima, Peru;; ^6^ New Jersey Medical School, Rutgers University, New Brunswick, NJ, USA;; ^7^ National Lung Hospital, Hanoi, Vietnam;; ^8^ FIND, Geneva, Switzerland;; ^9^ Medical University of South Carolina, Charleston, SC, USA.

**Keywords:** tuberculosis, TB screening, auscultation, respiratory sounds, artificial intelligence

## Abstract

**BACKGROUND:**

Improved TB screening requires non-invasive, low-cost, and rapid diagnostics. Digital stethoscopes utilising machine-learning approaches to analyse respiratory sounds have potential.

**METHODS:**

We assessed accuracy of a commercial digital stethoscope for TB diagnosis among TB symptomatic participants. The microbiological reference standard (MRS) was sputum TB-positive on either liquid culture, solid culture, or Xpert MTB/RIF Ultra. Adults were enrolled from South Africa, Uganda, Vietnam, and Peru, with pre-defined sampling of 60 MRS-positive and 180 MRS-negative participants over two stages. Respiratory sounds from six auscultation positions on the participant’s torso were analysed. The manufacturer (blinded to MRS status) provided participant scores and a test-positivity cut-off.

**RESULTS:**

Among 240 participants, 135 (56%) were female, 62 (26%) living with HIV, 35 (15%) current smokers, and 31 (13%) previously treated for TB. Estimates of sensitivity and specificity, adjusted for country-stratified sampling, were 77% (95% confidence interval [CI]: 65–85) and 50% (95% CI: 43–57), respectively. Sensitivity was lower among people living with HIV and those with sputum smear–negative TB and varied by country. Testing took 5 min per participant (median, interquartile range 4–6).

**CONCLUSION:**

These early data suggest that further refinement of this test is warranted. The device is simple to use, is inexpensive, and can be used offline.

Among the estimated 11 million people who develop TB every year, a quarter are never diagnosed and therefore do not receive adequate treatment, with consequent poor person-level outcomes and ongoing community transmission.^1^ To address this gap, there is an urgent need for new TB testing approaches, both for large-scale screening and for definitive diagnosis.^2,3^ Community-level screening needs to identify individuals at high risk for TB for subsequent confirmatory testing. For this purpose, tests need to be simple to administer, high throughput, low-cost, and with short turnaround time.^4,5^ For effective screening, a test should have higher sensitivity and specificity than symptom screening alone, and ideally be able to identify individuals with early or subclinical TB disease who are unlikely to present to health services.^6^ Traditional auscultation has long been used to examine patients for respiratory disease as it is non-invasive and gives information in real time. However, it is limited by its inherent subjectivity, given the reliance on the skill and experience of the clinician.^7^ However, a standardised system to analyse and interpret respiratory sounds has potential to increase utility, particularly with recorded sounds able to be analysed using artificial intelligence (AI) or machine-learning approaches.^7,8^ Such approaches, using digital stethoscopes, have primarily been used in respiratory conditions such as chronic obstructive pulmonary disease and pneumonia,^9,10^ with limited data available for TB screening.

We therefore aimed to assess the diagnostic utility of a commercially available digital stethoscope with AI predictive scoring for TB diagnosis among individuals with signs and symptoms of pulmonary TB.

## METHODS

This proof of principle study was conducted within the FEND-TB (Feasibility of Novel Diagnostics for TB in Endemic Countries) consortium (https://www.fend-tb.org/). FEND-TB includes a continuously enrolling protocol whereby adults (aged 18 and over) with presumptive TB are enrolled, after written informed consent, across research sites in four countries: South Africa, Uganda, Vietnam, and Peru. Testing of a digital stethoscope was conducted using a two-stage process.^11^ Stage 1 included testing of enrolled participants until 30 microbiological reference standard (MRS)-positive and 90 MRS-negative participants were recruited. Based on pre-specified sensitivity and specificity limits (correct classification of at least 22/30 MRS-positive and 47/90 MRS-negative participants), a decision was made whether to proceed to stage 2. Stage 2 included testing an additional 30 MRS-positive and 90 MRS-negative participants, randomly selected from the cohort of all participants who underwent lung auscultation, ensuring that participants from each of the four sites were included. The final study cohort included participants from both stages 1 and 2.

### Participant inclusion and specimen testing

Participants were included if there was clinical suspicion of active pulmonary TB, defined as cough ≥2 weeks and at least one other sign or symptom typical of active pulmonary TB (fever, night sweats, weight loss, fatigue, etc.). Participants who received any TB treatment in the 6 months prior to enrolment were excluded. Participants were recruited from either inpatient or outpatient health care settings. Collection of three sputum specimens for TB bacteriology testing was conducted over 2 days. The MRS was defined as *Mycobacterium tuberculosis* (MTB)-positive on any of liquid culture, solid culture, or Xpert MTB/RIF Ultra on any sputum specimen. MRS-negativity was defined as negative results on ≥2 liquid or solid culture tests, and Xpert MTB/RIF Ultra–negative if Xpert determinate results were obtained.

### Digital stethoscope

The wireless, handheld, digital stethoscope and accompanying AI predictive model (AI Diagnostics, South Africa, www.aidiagnostics.health) captured and analysed respiratory sounds from six auscultation positions on the torso of each participant (Supplementary Data Figure S1). These auscultation points were determined through discussion with the FEND-TB research team and AI Diagnostics based on the manufacturer’s early data and the need to limit the time taken to screen participants. Participants were instructed to breathe in and out for five breath cycles during recording by following video instructions on a laptop computer, which also recorded sounds. The system could be used offline, with data uploaded to the secure cloud storage when online.

### Digital stethoscope predictive scoring

Individual-level auscultation soundings were sent to the manufacturer for scoring. For stage 1 predictive scores, model predictions were updated three times by the manufacturer as the AI model was still under development, with final model scores used to decide progression to stage 2. Stage 2 data were analysed based on the last AI model from stage 1. Importantly, MRS status, TB diagnostic test results for determining MRS status, and sputum smear microscopy were not provided to the manufacturer throughout the study. The manufacturer provided the research team with individual participant probability scores, and whether audio artefacts were present for each participant. A cut-off value for a positive result was also provided by the manufacturer (≤0.608 for a negative result and >0.608 for a positive).

### Data analysis

Participants from both stages 1 and 2 were included in the primary analysis, including those with audio artefacts (supplementary analyses excluded those with artefacts). Sensitivity and specificity estimates were adjusted for stratified sampling by country, and 95% confidence intervals (CIs) were calculated using the stratified Wilson score method.^12^ Histograms and kernel density plots were estimated using the ggplot2 package for R, and receiver operating characteristic (ROC) curves and the area under the ROC curve (AUC) were estimated using the non-parametric trapezoidal rule using the pROC package for R (version 3.6.0).

### Ethical statement

The study was approved by ethics review boards in all participating sites and centrally by the Rutgers University Institutional Review Board (Human Research Protection Program Institutional Review Board, FWA00003913).

## RESULTS

**Figure 1.**
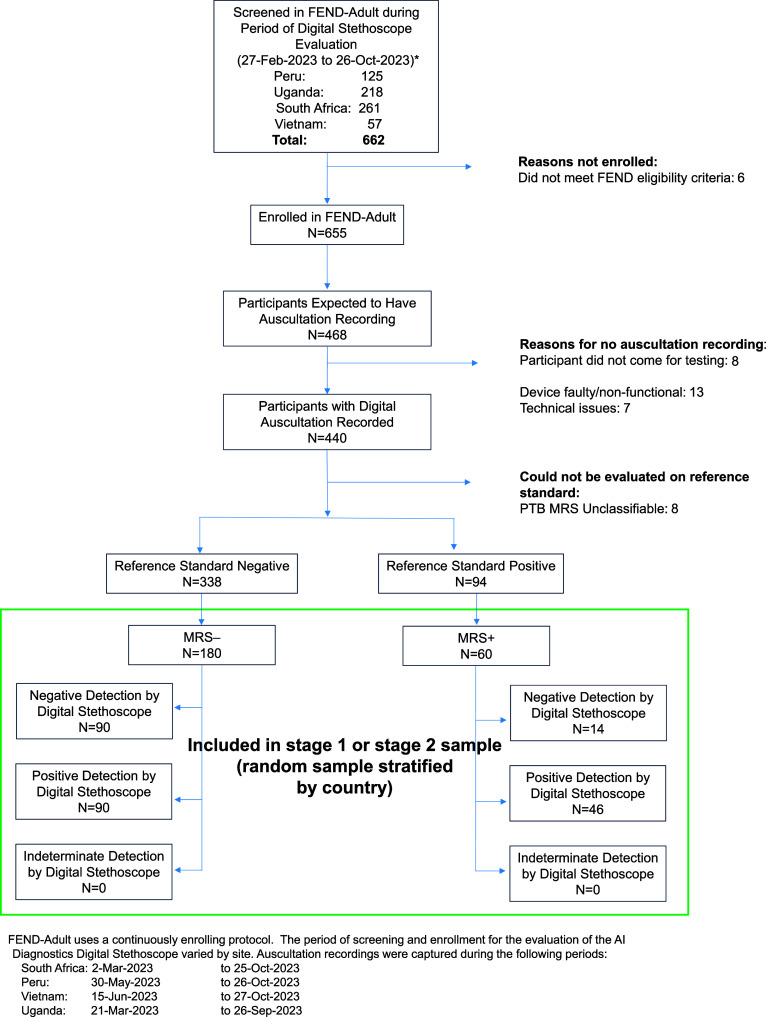
Flow diagram of participant inclusion.

Overall, 240 participants were included across the four sites (60 MRS-positive and 180 MRS-negative) (Figure 1; Table 1). These participants were recruited between 2 March 2023 and 27 October 2023, inclusive. The median age was 38 years, and 135 (56%) were female. Overall, 62 (26%) were living with HIV, with the majority of these participants from South Africa. Current daily smoking was reported by 35 (15%) participants, and 31 (13%) reported previous episodes of TB treatment. Among the 60 MRS-positive participants, 30 (50%) were sputum smear–positive. All 240 participants were classified as having determinate sound recordings. Sound artefacts were identified among 30 (13%) participants (12% and 13% MRS-positive and MRS-negative, respectively), and these included: excessive background noise, participant coughing during recording, stethoscope movement, or recorded speech during recording. Recordings took a median of 5 min (interquartile range 4–6). No adverse events were reported while performing the digital stethoscope procedure. Predictive score distributions of both MRS-positive and MRS-negative participants spanned the entire range of scores (Supplementary Data Figure S2). Above 0.608, digital stethoscope auscultation results were considered test positive.

**Table 1.** Participant characteristics at enrolment and MRS classifications based on sputum sampling performed at enrolment.

Characteristic	MRS-positive (N = 60)	MRS-negative (N = 180)	Total (N = 240)
Country, n (%)			
Peru	8 (13%)	22 (12%)	30 (13%)
South Africa	26 (43%)	90 (50%)	116 (48%)
Uganda	18 (30%)	47 (26%)	65 (27%)
Vietnam	8 (13%)	21 (12%)	29 (12%)
Age (years), median (IQR)	37 (25–45)	38 (27–50)	38 (27–47)
Female, n (%)	22 (37%)	113 (63%)	135 (56%)
Living with HIV, n/N (%)	17/60 (28%)^**A**^	45/179 (25%)	62/239 (26%)
Previous TB treatment, n (%)	8 (13%)	23 (13%)	31 (13%)
Smoking, n (%)			
Current daily	10 (17%)	25 (14%)	35 (15%)
Current, occasional	1 (2%)	1 (<1%)	2 (<1%)
Previous	5 (8%)	3 (2%)	8 (3%)
Never	44 (73%)	151 (84%)	195 (81%)
TB symptoms, n (%)			
Cough	60 (100%)	178 (99%)	238 (99%)
Fever	32 (53%)	95 (53%)	127 (53%)
Night sweats	34 (57%)	111 (62%)	145 (60%)
Weight loss	33 (55%)	77 (43%)	110 (46%)
Haemoptysis	8 (13%)	13 (7%)	21 (9%)
Other chronic lung conditions, n (%)	1 (2%)	9 (5%)	10 (4%)
Diabetes mellitus,^**B**^ n/N (%)	1/59 (2%)	9/180 (5%)	10/239 (4%)

MRS = TB microbiological reference standard; IQR = interquartile range; n = number positive; N = number with data.

A HIV status was unknown for one participant who was MRS-negative.

B No data on history of diabetes mellitus were obtained for one participant who was MRS-positive.

**Figure 2.**
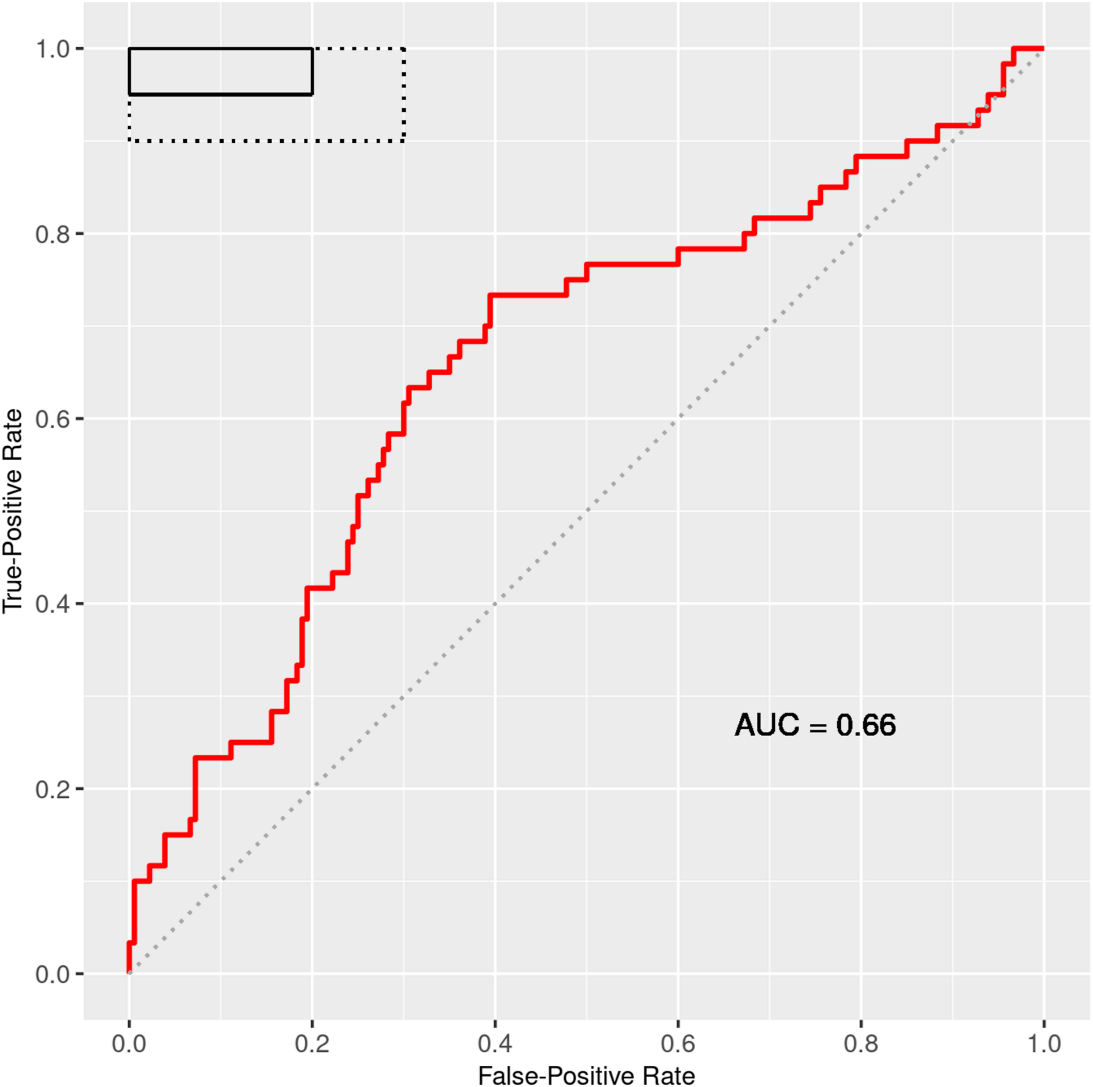
Receiver operating characteristic (ROC) curve and area under the curve (AUC) estimate for the total cohort. *The dotted line along the diagonal represents a test that is no better than chance. The solid box represents the optimal criteria of at least 95% sensitivity and 80% specificity, and the dashed box indicates the minimal criteria of at least 90% sensitivity and 70% specificity for a triage test.

The stage 1 sample primarily included participants from South Africa and Uganda and showed that the pre-specified stage 1 thresholds for sensitivity and specificity were met (Table 2). For the total cohort of 240 participants (stages 1 and 2 combined), the adjusted sensitivity estimate was 76.7% (95% CI: 65.1–85.2) and specificity estimate was 50.0% (95% CI: 43.0–56.9) (Table 2). Because of the updated AI model rounds during stage 1 and prior to stage 2, we also provide the results for stage 2 in Table 2. Sensitivity and specificity estimates did not change significantly when artefacts were excluded. The ROC AUC was estimated at 0.66 (95% CI: 0.57–0.74) (Figure 2).

**Table 2.** Sensitivity and specificity estimates for stage 1, stage 2, and total cohort.

	Primary outcome			Excluding artefacts		
	N	n	Estimate (95% CI)^**A**^	N	n	Estimate (95% CI)^**A**^
Stage 1^**B**^						
Sensitivity	30	26	86.7% (63.6–95.8)	27	24	88.9% (64.7–97.2)
Specificity	90	49	54.4% (44.8–64.3)	80	45	56.3% (46.0–66.5)
Stage 2						
Sensitivity	30	20	66.7% (49.2–80.2)	26	18	69.2% (50.4–82.6)
Specificity	90	41	45.6% (36.3–55.2)	77	35	45.5% (35.6–55.5)
Total cohort (stage 1 and stage 2 combined)						
Sensitivity	60	46	76.7% (65.1–85.2)	53	42	79.2% (67.0–87.5)
Specificity	180	90	50.0% (43.0–56.9)	157	80	51.0% (43.5–58.2)

N = total included; n = number correctly identified on digital stethoscope; CI = confidence interval.

A Stratified Wilson score confidence interval.

B Pre-specified levels for continuing from stage 1 to stage 2 were sensitivity 22/30 and specificity 47/90.

Although the sample sizes were small when the cohort was divided by participant characteristics, there appears to be variation in sensitivity by country, with point estimates from Peru and Vietnam showing lower sensitivity as compared with South Africa and Uganda. Not surprisingly, sensitivity was higher among HIV-negative and sputum smear–positive participants (Table 3).

**Table 3.** Sensitivity and specificity estimates by participant characteristics (artefacts included).

	N	n	Sensitivity (95% CI)^**A**^	N	n	Specificity (95% CI)^**A**^
Country						
Peru	8	5	62.5% (30.6–86.3)	22	13	59.1% (38.7–76.7)
South Africa	26	19	73.1% (53.9–86.3)	90	50	55.6% (45.3–65.4)
Uganda	18	17	94.4% (74.2–99.0)	47	13	27.7% (16.9–41.8)
Vietnam	8	5	62.5% (30.6–86.3)	21	14	66.7% (45.4–82.8)
HIV status						
Living with HIV	17	12	70.6% (40.8–86.6)	45	25	55.6% (41.2–69.1)
HIV negative	43	34	79.1% (65.7–88.2)	134	65	48.5% (40.2–56.9)
Sputum smear status						
Positive	30	26	86.7% (68.0–94.6)	1	3	33.3% (6.1–79.2)
Negative	30	20	66.7% (49.2–81.6)	89	177	50.3% (43.0–57.6)
Previous TB						
Yes	8	7	87.5% (37.9–97.7)	23	12	52.2% (33.0–70.8)
No	52	39	75.0% (62.8–84.3)	157	78	49.7% (42.0–57.4)

N = total included; n = number correct on digital stethoscope; CI = confidence interval.

A Estimates use weights proportional to the sample size in a country within the microbiological reference standard (MRS)-positive or MRS-negative category and are accompanied by stratified Wilson’s confidence intervals. No adjustment was done for country-specific estimates.

## DISCUSSION

These data demonstrate the potential for use of a digital stethoscope for initial screening for TB. The device was simple to use, was rapid, and could be used offline. The World Health Organization outlines minimal requirements for a screening test of >90% sensitivity and >70% specificity.^4^ While the estimates of sensitivity (77%) and specificity (50%) shown here fall short of these targets, more recent modelling suggests that non-sputum screening tests, such as a digital stethoscope, with lower sensitivities (from 70%) could still result in improved case detection if accessibility is increased.^13^ A digital stethoscope has the potential to be used both for active, community TB screening outside of health facilities and within facilities where access to laboratory facilities is limited, thereby allowing for improved screening accessibility. Decreased cost is also a key factor in improving screening access. The test developer currently estimates that screening will cost USD0.67 per test, with capacity to reduce cost further when utilised in a high throughput screening programmatic activity.

While the sensitivity shown here is above the 70% threshold that may be acceptable, it is possible that specificity will further improve if the test is performed among a population not already under investigation for TB (i.e., less likely that another respiratory disease will be identified as TB). Based on current guidance, all the participants included here should be tested for TB using a rapid molecular test. Given the extent of respiratory symptoms in this cohort, it is likely that there were other respiratory conditions that could have confounded digital stethoscope readings. Screening of an otherwise well population at the community level might, therefore, improve specificity, while also providing an opportunity to identify other non-TB respiratory conditions.^2^

During this proof of principle study, the AI model used to generate individual-level probabilities was refined several times to improve prediction. Additionally, the training data used by the manufacturer for the AI model were generated using an earlier prototype version of the digital stethoscope, only from South African participants and only included sound recordings. At the time of the study, no clinical data (such as HIV status or symptoms) were used to train the AI model. Therefore, it is possible that further AI model training with larger and more diverse training datasets drawn from the updated design-locked digital stethoscope version and the inclusion of simple clinical data may improve screening accuracy. In this study, the inclusion of primarily African participants in stage 1 and sole use of South African data by the manufacturer for model development may have resulted in the better sensitivity and specificity estimates in stage 1, compared with stage 2, which included participants from more diverse geographical locations, including Peru and Vietnam. Similarly, we observed some differences in sensitivity across the four countries included in this study. While, the sample size was small, these differences suggest that prediction models may need to be trained in different settings, or that thresholds for prediction might need to be varied across settings and countries. Overall, as was the case for AI-assisted reading of digital chest X-rays for TB diagnosis, ongoing refinement of prediction models using machine learning and based on large datasets has potential to improve utility over time.^14^

There are other possible advantages of using a digital stethoscope for screening. While ultra-portable digital chest X-ray machines have now been developed, they remain expensive and likely require significant ongoing maintenance, with consequent constraints on accessibility and scale-up.^2^ In contrast, digital stethoscopes could be deployed and scaled up for community screening at multiple locations simultaneously. By enabling the screening of a larger number of community individuals due to improved scalability, digital stethoscopes may still gain diagnostic yield, even with more limited sensitivity.^13^ Additionally, X-ray screening during pregnancy, a population where improved TB screening is urgently needed,^15^ often raises concerns, whereas a digital stethoscope is likely to be acceptable.

## CONCLUSION

These early data suggest that screening with a digital stethoscope may provide improved sensitivity and specificity over that provided by symptom screening alone, where sensitivity ranges from 42% to 71% and specificity from 65% to 95%.^16^ It is also possible that combining digital stethoscope screening results with the presence of symptoms might further improve sensitivity, as is the case for digital X-ray.^16^ This suggests that further development and refinement of a digital stethoscope and accompanying AI-based model is warranted. We would recommend that further testing occur among both participant populations with a high likelihood of TB (such as in our study) and broader populations. We would also suggest a comparison with prediction data from digital chest X-rays in the same populations, given that development of these systems is substantially more advanced. Finally, incorporation of respiratory vibration recording might also improve screening accuracy.^17^

## Supplementary Material


