# Supplementary material for: Diagnostic accuracy of a commercial AI digital stethoscope for diagnosis of TB
Source: IJTLD Open. 2025 Oct 10;2(10):610–5. doi: 10.5588/ijtldopen.25.0360 (PMC12517259; doi:10.5588/ijtldopen.25.0360)

Supplementary information

Supplementary figure 1: Digital stethoscope equipment and auscultation positions for recording with the digital stethoscope.

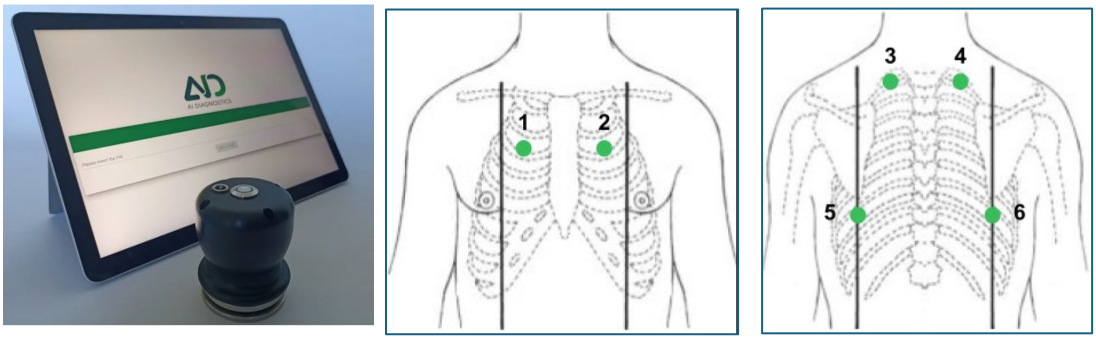

Supplementary figure 3: Histograms and smoothed kernel density estimates of digital stethoscope probability scores stratified by MRS status.

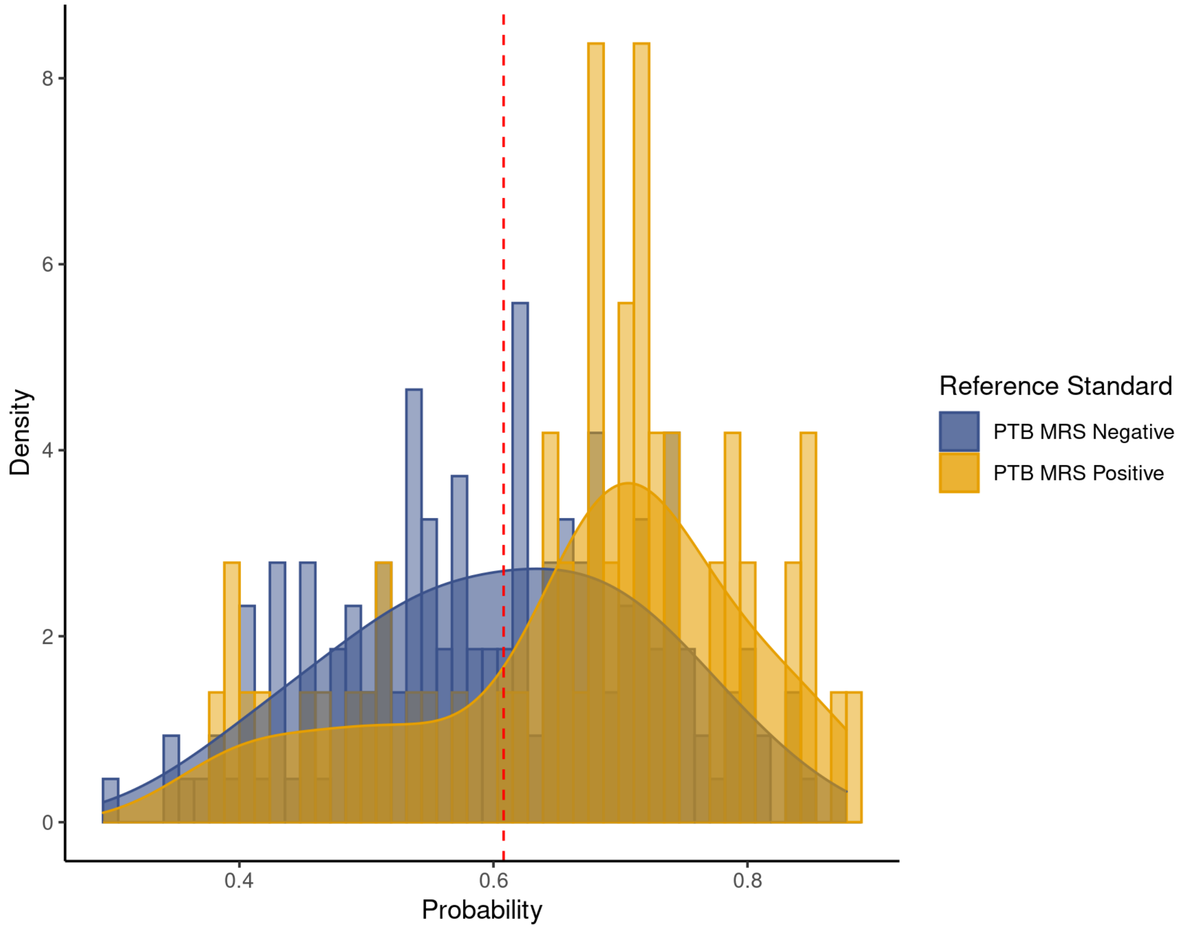

Supplement: Supplementary file 1 [file ijtldopen25-0360_supplementarydata1.pdf]
